# The Impact of Divorce on Intergenerational Support for Older Adults in China

**DOI:** 10.1093/geroni/igaf122.2357

**Published:** 2025-12-31

**Authors:** Tianqi Zhou, Merril Silverstein, Ying Xu, Xiaoyu Fu

**Affiliations:** Syracuse University, Syracuse, New York, United States; Syracuse University, Syracuse, New York, United States; Syracuse University, Syracuse, New York, United States; Syracuse University, Los Angeles, California, United States

## Abstract

Studies of the long-term effects of parental divorce on intergenerational relationships in later life suggest that divorce may weaken parent-child ties, reducing the likelihood of support from children. However, alternative theories propose that the disadvantaged status of divorced parents may prompt greater support, particularly in a familistic culture that prioritizes intergenerational responsibility. This study examines the relationship between parental marital status and financial and instrumental support provided by adult children in China. Using data from the 2022 Chinese Family Panel Studies, we analyze responses from 2,088 adult children with living fathers and 2,198 with living mothers, both parents aged 60+. Logistic regression models reveal that adult children are more likely to provide financial support to divorced mothers than to married mothers, while no such effect is observed for divorced fathers. Divorced parents, irrespective of sex, are more likely to receive instrumental support, such as help with household chores. Notably, divorced mothers and fathers are no less likely to receive either type of support than their widowed counterparts despite the former’s greater emotional distance from children. Our study extends research on the relationship between marital status and support in societies with strong filial norms; family support is more likely to be directed toward vulnerable unmarried older parents whose financial and caregiving needs cannot be met by spouses, regardless of how their unmarried status was achieved. These findings highlight the enduring strength of filial responsibility in China, demonstrating its prominence over emotional closeness in shaping intergenerational support.

